# A novel mutation in DDR2 causing spondylo-meta-epiphyseal dysplasia with short limbs and abnormal calcifications (SMED-SL) results in defective intra-cellular trafficking

**DOI:** 10.1186/1471-2350-15-42

**Published:** 2014-04-11

**Authors:** Adila Al-Kindi, Praseetha Kizhakkedath, Huifang Xu, Anne John, Abeer Al Sayegh, Anuradha Ganesh, Maha Al-Awadi, Lamya Al-Anbouri, Lihadh Al-Gazali, Birgit Leitinger, Bassam R Ali

**Affiliations:** 1 Department of Genetics, Sultan Qaboos University Hospital, Sultan Qaboos University, Al koudh, 123, Muscat Sultanate of Oman; 2 Department of Pathology, College of Medicine and Heath Sciences, United Arab Emirates University, P.O. Box 17666 Al Ain, United Arab Emirates; 3 National Heart and Lung Institute, Imperial College London, London SW7 2AZ, United Kingdom; 4 Pediatric Ophthalmology, Sultan Qaboos University Hospital, Sultan Qaboos University, Alkoudh, 123, Muscat, Sultanate of Oman; 5 Department of Paediatrics, College of Medicine and Heath Sciences, United Arab Emirates University, Al Ain, United Arab Emirates

**Keywords:** DDR2, Spondylo-meta-epiphyseal dysplasia, Trafficking defect, SMED-SL, ERAD, Optic atrophy

## Abstract

**Background:**

The rare autosomal genetic disorder, Spondylo-meta-epiphyseal dysplasia with short limbs and abnormal calcifications (SMED-SL), is reported to be caused by missense or splice site mutations in the human discoidin domain receptor 2 (*DDR2*) gene. Previously our group has established that trafficking defects and loss of ligand binding are the underlying cellular mechanisms of several SMED-SL causing mutations. Here we report the clinical characteristics of two siblings of consanguineous marriage with suspected SMED-SL and identification of a novel disease-causing mutation in the *DDR2* gene.

**Methods:**

Clinical evaluation and radiography were performed to evaluate the patients. All the coding exons and splice sites of the *DDR2* gene were sequenced by Sanger sequencing. Subcellular localization of the mutated DDR2 protein was determined by confocal microscopy, deglycosylation assay and Western blotting. DDR2 activity was measured by collagen activation and Western analysis.

**Results:**

In addition to the typical features of SMED-SL, one of the patients has an eye phenotype including visual impairment due to optic atrophy. DNA sequencing revealed a novel homozygous dinucleotide deletion mutation (c.2468_2469delCT) on exon 18 of the *DDR2* gene in both patients. The mutation resulted in a frameshift leading to an amino acid change at position S823 and a predicted premature termination of translation (p.S823Cfs*2). Subcellular localization of the mutant protein was analyzed in mammalian cell lines, and it was found to be largely retained in the endoplasmic reticulum (ER), which was further supported by its N-glycosylation profile. In keeping with its cellular mis-localization, the mutant protein was found to be deficient in collagen-induced receptor activation, suggesting protein trafficking defects as the major cellular mechanism underlying the loss of DDR2 function in our patients.

**Conclusions:**

Our results indicate that the novel mutation results in defective trafficking of the DDR2 protein leading to loss of function and disease. This confirms our previous findings that DDR2 missense mutations occurring at the kinase domain result in retention of the mutant protein in the ER.

## Background

Spondylo-meta-epiphyseal dysplasia (SMED), short limb-hand type (SMED-SL, OMIM 271665) is a rare autosomal recessive disorder affecting human skeletal growth. The condition is characterized by disproportionately short stature, platispondyly, abnormal epiphyses and metaphyses, shortening of the lower and upper limbs, short broad fingers and punctate calcifications [1-4]. This bone dysplasia is progressive, with serious complications leading to death in some cases. Atlantoaxial instability resulting in cord damage has been the most reported cause of death [2,5]. Homozygous mutations occurring in the Discoidin domain receptor 2 gene (*DDR2*, MIM 191311) have been identified as the cause for this severely dwarfing condition [6,7]. The *DDR2* gene encodes one of the two members of a unique receptor tyrosine kinase (RTK) subfamily known as the discoidin domain containing receptors (DDRs), which recognize collagen as their ligands [8,9]. Upon collagen binding, the receptor displays delayed and sustained tyrosine phosphorylation which further elicits downstream signalling to cellular metabolic pathways that cross-talk at various points [10,11]. In addition, dysregulation of DDR2 has been shown to be associated with various human diseases such as fibrosis, arthritis and cancer and like other RTKs, DDRs are emerging as potential therapeutic targets [12].

DDR2 is commonly expressed in cells of mesenchymal origin and is activated by fibrillar collagens [8,9] and collagen X [13]. DDR2 has been shown to play a critical role in cell invasion and collagen remodelling through the regulation of matrix metalloproteases and collagen fibrillogenesis [10,14-16]. The involvement of DDR2 in skeletal growth was demonstrated by the DDR2 knockout mice, which display skeletal abnormalities that reflect the SMED-SL phenotype of humans [17]. The abnormal skeletal development in DDR2 deficient mice was due to reduced chondrocyte proliferation in the growth plate. A spontaneous, autosomal recessive mutation in a mouse colony was characterised that resulted from deletion of most of the *Ddr2* gene [18]. These mutant mice were also found to be infertile due to gonadal dysfunction including impaired spermatogenesis and ovulation, which were attributed to defects in overall endocrine function [18-20].

DDRs are plasma membrane RTKs comprising an extracellular domain (ECD), a transmembrane domain (TM), a large cytosolic juxtamembrane domain and a C-terminal catalytic tyrosine kinase domain. The ECD is necessary and sufficient for ligand binding and consists of an N-terminal discoidin homology (DS) domain followed by a region unique to the DDRs that contains a DS-like domain [21-23]. The DDRs exist as preformed homodimers on the cell membrane even in the absence of collagen [24,25]. The exact signaling pathways and binding partners through which DDR2 controls bone growth remain unknown despite significant progress made in recent years in understanding the structural basis of collagen recognition [26]. In recent reports it was shown that DDR2 modulates the phosphorylation of Runx2, a master transcription factor involved in skeletal development [27,28]. In addition, DDR2 has been shown to mediate the secretion of lysyl oxidase [29], an enzyme that catalyzes cross-linking of collagen fibers, an essential modification to strengthen bone.

A recent study has shown that the DDR2 ECD aids the formation of mineralized calcium deposits *in vitro*, and this activity is independent of the tyrosine kinase activity [16]. The authors proposed that the presence of DDR2 ECD in the mutant proteins along with impaired signaling leading to increased calcification could be a potential mechanism in SMED-SL. Mutations prevalent in SMED-SL disorder have been mapped to the extracellular domain and tyrosine kinase domain of DDR2 [6,7]. Recently we have shown that DDR2 missense mutations occurring at the kinase domain (p.T713I, p.I176R, p.R752C) result in retention of the mutant protein in the endoplasmic reticulum (ER), while the ectodomain mutant (p.E113K) is expressed on the cell surface, but failed to bind to collagen, thus elucidating two different cellular mechanisms resulting in loss of function of the protein leading to disease [7].

ER retention of misfolded proteins and subsequent degradation (ERAD) is a recurring theme in many skeletal disorders [30-33] and numerous other monogenic diseases [31,34-36]. Inhibition of ERAD coupled with proteostasis modulation has been shown to enhance folding, trafficking and activity of unstable proteins and is emerging as a potential strategy to combat many protein misfolding disorders [37]. Defining the potential contribution of specific mutations and intracellular stress to the pathophysiology of the disease will pave avenues for targeted therapeutic intervention and disease management in the future.

We report here the clinical and molecular findings in SMED-SL patients of consanguineous origin from Oman and the identification of a novel disease-causing truncating mutation in DDR2. The sub-cellular localization and functional status of the mutant and wild type proteins were compared in a mammalian expression system. Our results indicate that the novel mutation results in defective trafficking of the protein and loss of its activation by collagen.

## Methods

### Clinical evaluations

This study complies with the Helsinki Declaration and has been approved by Al-Ain District Human Research Ethics Committees (protocol No. AAMD/HREC 10/09) and the family provided an informed consent. Clinical assessment was performed by a clinical geneticist and the radiography imaging of the subjects was also evaluated.

### Chemicals and reagents

Collagen I (acid-soluble from rat tail) was from Sigma (Gillingham, UK). The antibodies and their sources were as follows: antibodies for immunofluorescence: mouse anti-HA-tag monoclonal antibody (dilution 1:200; Cell Signaling Technology), rabbit anti-calnexin polyclonal antibody (dilution 1:200, Santa Cruz), Alexa Fluor 568-goat anti-mouse IgG (dilution 1:200; Molecular Probes), Alexa Fluor 568-goat anti-rabbit IgG (dilution 1:200; Molecular Probes), Alexa Fluor 488-goat anti mouse IgG (dilution 1:200, Molecular Probes). Antibodies for Western blotting: goat anti-DDR2 from R & D Systems (Abingdon, UK); mouse anti-phosphotyrosine, clone 4G10, from Upstate Biotechnology (Lake Placid, NY); sheep anti-mouse Ig-horseradish peroxidase (Amersham Biosciences UK, Chalfont St Giles, UK); rabbit anti-goat Ig-horseradish peroxidase (Zymed Laboratories, San Francisco, CA).

### Mutation screening

Genomic DNA was extracted from peripheral leukocytes using the Flexigene DNA kit (Qiagen). The coding exons and exon-flanking sequences were analyzed by PCR (Taq polymerase, Qiagen) followed by direct sequencing using the BigDye Terminator v3.1 Cycle Sequencing kit (Applied Biosystems, Foster City, CA). Capillary electrophoresis was performed on an ABI PRISM 3130xl DNA Analyzer (Applied Biosystems). Primers and PCR conditions for amplification of the coding exons (Exons 3–18) and splice sites have been described previously [7]. Sequences were aligned to the NCBI reference sequence NM_001014796, using ClustalW2 algorithm and mutations were designated with reference to the translation start site.

### Generation of mammalian expression constructs

The frame-shift mutation, c.2468_2469delCT was introduced into the untagged expression vector, pcDNA3.1-DDR2 [22] by site directed mutagenesis using *Pfu* Ultra HF polymerase (Stratagene, La Jolla, CA). The primers used were: DDR2-S823Cfs*-F: CATTTGTCCTGACTGTGTATAAGCTGATG, DDR2-S823Cfs*-R: CATCAGCTTATACACAGTCAGGACAAATG. This construct was subsequently used for performing deglycosylation and receptor binding assays. An HA tag was inserted prior to the stop codon (introduced by the frame-shift mutation) of the mutated construct, using two cycles of site directed mutagenesis. In the first cycle, the initial five amino acids (YPYDV) of the HA tag were introduced at the C-terminus of the mutated *DDR2*, using the primers: DDR2_Mut2A_FP: GTCCTGACTGTGTATACCCATACGATGTTTAAGCTGATGCTCAGCTG, DDR2_Mut2A_RP: CAGCTGAGCATCAGCTTAAACATCGTATGGGTATACACAGTCAGGAC. The remaining four amino acids (PDYA) of the tag were introduced to the above construct using the following primers: DDR2_Mut2B_FP: ATACCCATACGATGTTCCAGATTACGCTTAAGCTGATGCTCAGC, DDR2_Mut2B_RP: GCTGAGCATCAGCTTAAGCGTAATCTGGAACATCGTATGGGTAT. All the plasmids have been sequenced to confirm the introduction of intended changes.

### Cell culture and transfection

HeLa cells were cultured in DMEM (Invitrogen, Carlsbad, CA) supplemented with 10% fetal bovine serum, 2 mM L-glutamine and 100 U/ml penicillin/streptomycin at 37°C with 5% CO2. For localization experiments, cells were grown on sterile cover slips in 24-well tissue culture plates and transient transfection was performed by FuGENE HD transfection reagent according to the manufacturer’s instructions (Promega, Madison, WI) using 0.5 μg plasmid DNA. GFP-H-Ras plasmid was used as a plasma membrane marker and co-transfected with HA-tagged wild type or mutant plasmids. The cells were processed for staining and imaging after 24 hours of transfection.

Human embryonic kidney (HEK-293) cells (ATCC, Manassas, VA, USA) were cultured in Dulbecco’s modified Eagle’s medium/F12 medium (Invitrogen) supplemented with 10% fetal bovine serum (Invitrogen), penicillin (10 U/ml) and streptomycin (100 μg/ml) at 37°C with 5% CO2. For transfection, cells were grown in 24-well tissue culture plates and were transfected with untagged DDR2 wild-type or mutant plasmid DNA using calcium phosphate precipitation.

### Immunocytochemistry and imaging

Twenty-four hours after transfection, HeLa cells grown on cover slips were washed with phosphate-buffered saline (PBS) and fixed by methanol at 22°C for 5 min. Fixed cells were washed in PBS three times and blocking was carried out in 1% BSA (Sigma) in PBS for 30 min at room temperature. After blocking, the cells were incubated with either mouse monoclonal anti-HA antibody alone or co-stained with both mouse monoclonal anti-HA antibody and rabbit polyclonal anti-calnexin antibodies, for 45 min at room temperature. The cells were washed with PBS, and incubated with the respective secondary antibodies for 45 min at room temperature, washed several times with PBS and mounted in immunofluor medium (ICN Biomedicals). Confocal microscopy and imaging was performed with a Nikon Eclipse system (Nikon Instruments Inc., Melville, NY).

### Collagen-induced DDR2 autophosphorylation

The assay was performed as described previously in details [22]. Twenty-four hours after transfection, HEK-293 cells were incubated with serum-free medium for 16 h. Cells were then stimulated with collagen I (5–50 μg/ml) for 90 min at 37°C. Cells were lysed in 1% Nonidet P-40, 150 mM NaCl, 50 mM Tris, pH 7.4, 1 mM EDTA, 1 mM phenylmethylsulfonyl fluoride, 50 μg/ml aprotinin, 1 mM sodium orthovanadate and 5 mM NaF. Aliquots of the lysates were analyzed by SDS–PAGE followed by blotting onto nitrocellulose membranes. The duplicate blots were probed with either anti-phosphotyrosine monoclonal antibody or anti-DDR2 antibodies followed by corresponding horseradish peroxidise-conjugated secondary antibodies. Detection was performed using Pierce ECL 2 Western Blotting substrate (Thermo Scientific) and an Ettan DIGE Imager (GE Healthcare Biosciences).

### Endoglycosidase H deglycosylation assay

Forty eight hours after transfection, HEK-293 cells were lysed as described above. Twenty microliter of each cell lysate was denatured in 1× glycoprotein denaturing buffer (0.5% SDS and 1% β-mercaptoethanol) for 5 min at 100°C. The denatured lysates were then split into two equal aliquots which were incubated for 3 h at 37°C in the presence or absence of 10 U of Endoglycosidase H (New England Biolabs). Samples were then resolved on 7.5% SDS–PAGE followed by blotting onto nitrocellulose membranes and probing with anti-DDR2 antibodies, as described above.

## Results

### Clinical characteristics of SMED-SL patients

**Figure 1:**
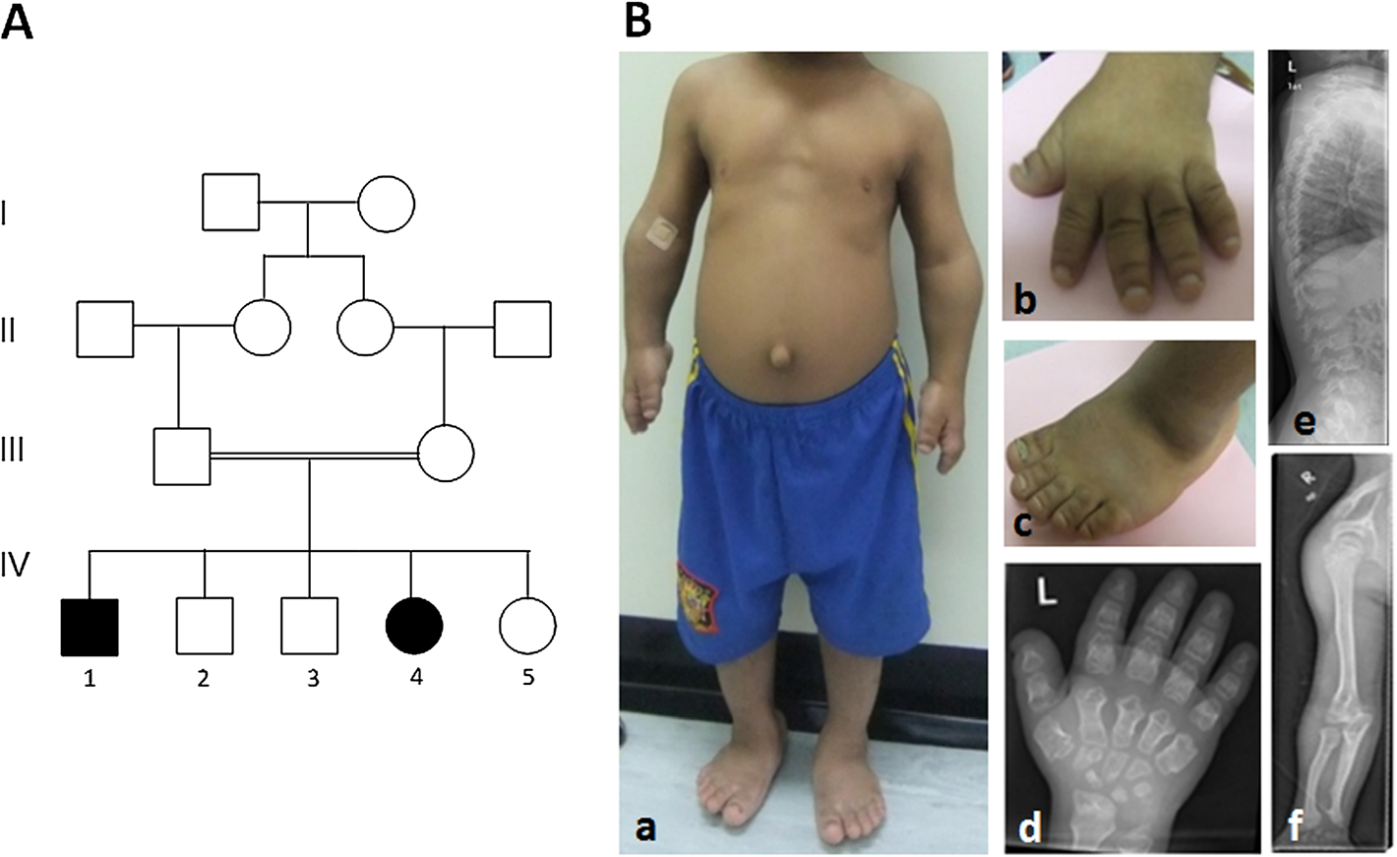
**Pedigree, dysmorphic features and radiographic findings in the affected family. (A)** Pedigree of the consanguineous Omani family. The family displays typical recessive modes of inheritance. Double bar represents consanguinity. Affected members are represented by filled symbols. **(B)** Dysmorphic features **(a)** 10 year old affected boy, note short limbs, narrow chest and protuberant abdomen with everted umbilicus **(b) **&**(c)** short broad hands and feet with stubby fingers and toes with short hypoplastic nails **(d)** X-ray of the left hand at 10 years of age showing short and broad tubular bones with diaphyseal constriction, narrowing of the metacarpal bones proximally, triangular distal phallanges, and irregular epiphyses which are cone shaped. L on the upper left corner of the X-ray refers to left hand **(e)** Lateral X-ray of the spine showing platyspondyly with pear shaped vertebrae, some showing anterior beaking, irregularities of the vertebral endplated and punctate calcifications **(f)** X-ray of the upper arm at 10 years of age showing mesomelic shortening of the long bones, madelung deformity, and irregular distal epiphyses of radius and ulna.

The parents are first cousins of Omani origin (Figure 1A). The two affected children, a boy and a girl, were assessed, aged 10 years and 7 years. Both presented with severe short stature, height well below 3^rd^ centile (-6SD-7SD) with relative macrocephaly, wide anterior fontanelle and partial alopecia at the vertex. There was shortening of all limbs, particularly the distal segments, madelung deformity of the forearm with limited extension of the elbow joints, and bowing of the tibias. The hands and feet were very small with short stubby fingers and toes with hypoplastic nails (Figure 1B panels (b) and (c)). The chest was narrow with pectus excavatum and there was thoracic scoliosis with lumber gibbus. The children had coarse facial features with midface hypoplasia, hypertelorism, infraoribital fullness with creases, short flat nose with wide anteverted nares, long philtrum with prominent full lower lip, misaligned teeth and micrognathia. Both had moderate conductive hearing loss due to glue ear. The girl had progressive visual impairment due to bilateral optic atrophy. Both children have normal intelligence. Both parents, maternal and paternal grandmother are said to be short but no measurements were available.

Skeletal survey on both children revealed changes typical of SMED-SL including short and broad long bones with irregular, flared metaphyses of the proximal and distal ends with irregular trabecular structure (Figure 1B-f). In the spine there was platyspondyly, pear shaped vertebrae, anterior beaking, irregular endplates and punctate calcification (Figure 1B-e). There was a small thoracic diameter with short broad ribs. The tubular bones of the hands were very short and broad with diaphyseal constriction (Figure 1B-f). The metacarpal bones were narrower at the proximal than distal ends giving the appearance of drumsticks and the distal phalanges were triangular in shape (Figure 1B-d).

### The patients have a homozygous truncating mutation in the *DDR2* gene

**Figure 2:**
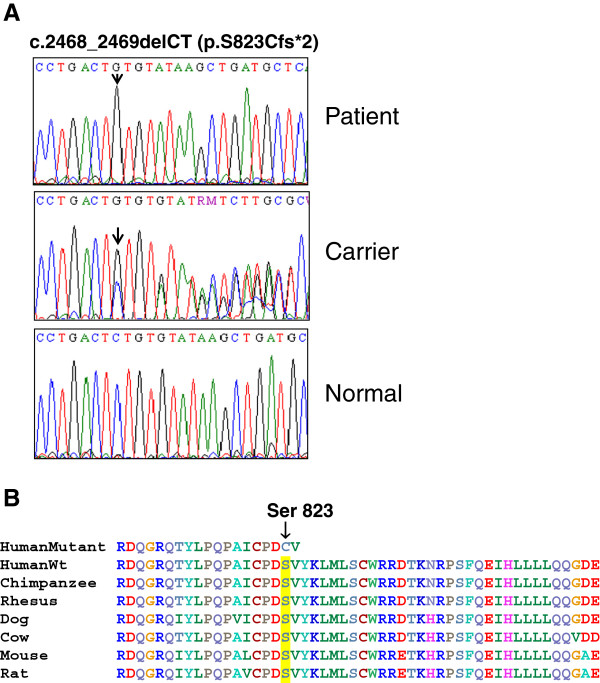
**Identification of a novel dinucleotide deletion in the SMED-SL patient.** Direct sequencing analysis was carried out for all the coding exons of the *DDR2* gene. **(A)** This panel shows a representative chromatogram of the DNA sequences indicating the c.2468_2469delCT mutation in the affected individuals and their parents. Arrows indicate the position of the c.2468_2469delCT mutation in the patient and one of the heterozygous carriers **(B)** Multiple sequence alignment of mutant and wild type DDR2 proteins from different species. The S823 residue is highlighted.

The genomic DNA from the parents and the male patient was isolated and used as template to amplify all the *DDR2* gene coding exons and splice sites. Direct sequence analysis of the PCR amplicons revealed a homozygous two nucleotide deletion in exon 18 (c.2468_2469delCT) of the patient (Figure 2A). In addition to causing an amino acid substitution, S823C, the deletion leads to a frame-shift followed by a stop codon two amino acid residues downstream of the mutation (p.S823Cfs*2). Both parents were found to be heterozygous for the mutation (Figure 2A). The mutation is predicted to result in a truncated DDR2 protein lacking the C-terminal 32 amino acids of the cytoplasmic tyrosine kinase domain. The serine residue at position 823 and the amino acid residues in the truncated region of the mutant are highly conserved across various species (Figure 2B). This mutation has not been previously reported in any of the SNP or clinical variant databases. Mutation prediction programmes (Mutation taster) predicted that these changes are highly likely disease-causing by affecting the protein structure and/or function. Additionally, truncating mutations occurring in the last exon of a gene are not suppressed by nonsense mediated mRNA decay (NMD) and might exert a dominant negative effect [38].

### The mutation leads to defective intracellular targeting of the mutant protein

**Figure 3:**
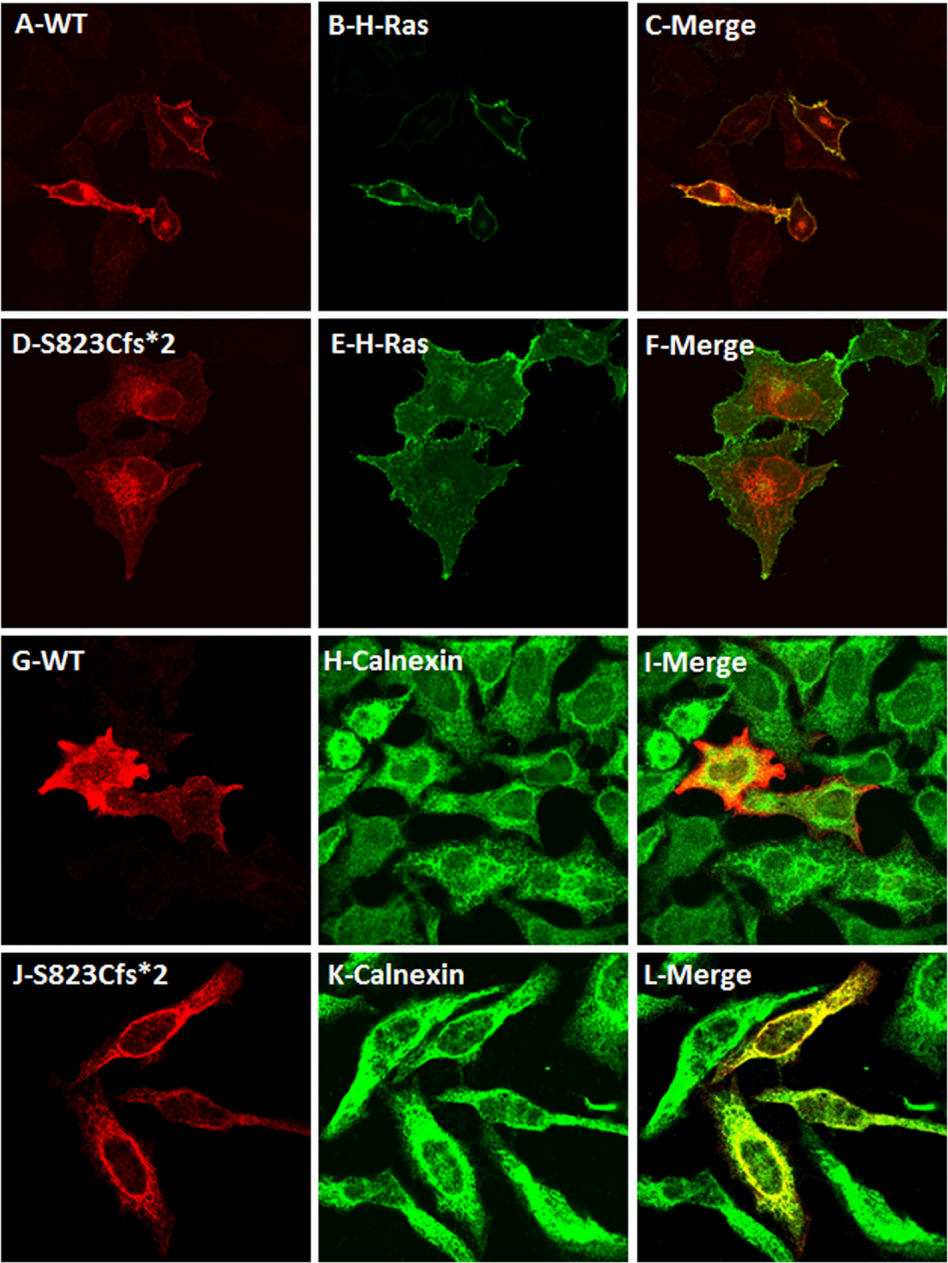
Comparison of intracellular localization of DDR2 wild type and S823Cfs* mutant in relation with plasma membrane marker H-Ras and ER marker calnexin. HeLa cells were co-transfected with C-terminally HA-tagged wild type or mutant DDR2 and EGFP tagged H-Ras constructs and stained with anti-HA antibodies. **(A)** and **(D)** shows the localization patterns of WT and the mutant DDR2 proteins. **(B)** and **(E)** shows the localization of the marker protein H-Ras to the plasma membrane. **(C)** shows co-localization of WT-DDR2 with H-Ras and **(F)** shows exclusion of S823Cfs*-DDR2 from the plasma membrane. **(G)** and **(J)** show distribution of HA-tagged wild type DDR2 and S823cfs* mutant in HeLa cells transfected with the indicated construct. **(H)** and **(K)** show the localization of the ER marker protein calnexin. **(I)** and **(L)** show the extent of co-localization of DDR2 proteins with calnexin. For presentation purpose, images **(G)**-**(L)** were pseudocolored as either red (DDR2) or green (calnexin) using ImageJ software.

Previously we reported that missense mutations located on the intracellular kinase domain of DDR2 resulted in the ER retention and loss of activation of the protein. To investigate the implications of the novel disease-causing mutation (S823Cfs*) on the DDR2 protein, we generated by site directed mutagenesis, the mutant cDNA fused to an HA epitope tag at the C-terminus and expressed it in a mammalian expression system. The subcellular localization of the mutant protein was determined by immunohistochemical staining and confocal imaging of HeLa cells expressing the mutant protein. As expected, the wild type DDR2 was found to localize primarily to the plasma membrane and co-localize with the plasma membrane protein H-Ras tagged with GFP (Figure 3, panels A,B and C). On the contrary, the localization pattern of the S823Cfs* mutant was distinct from that of the wild type protein. As shown in Figure 3 (panels D,E and F), the mutant protein was found to localize predominantly in the endoplasmic reticulum, along with calnexin. A fraction of the wild type protein was also found to co-localize with the ER marker calnexin (Figure 3, panels G,H and I); this is presumably the in transit biosynthetic fraction of the protein or the fraction that failed the stringent ER quality control machinery. In cells co-transfected with GFP-H-Ras and the S823Cfs* mutant, the fluorescence from the mutant was found to be excluded from the plasma membrane (Figure 3, panels J,K and L).

### Incomplete glycosylation and loss of activation of the disease-causing mutant

In all the previously reported SMED-SL missense mutants of DDR2, collagen mediated activation was disrupted due to either mis-folding and trafficking defects or loss of ligand binding. The new disease-causing mutation in this report is predicted to result in a truncated protein which lacks the last 32 amino acid residues of the cytoplasmic tyrosine kinase domain. To determine whether the novel disease-causing mutant was functionally active *in vivo*, we expressed the mutant and wild-type DDR2 in HEK-293 cells and analyzed their N-glycosylation status and activation by collagen.

**Figure 4:**
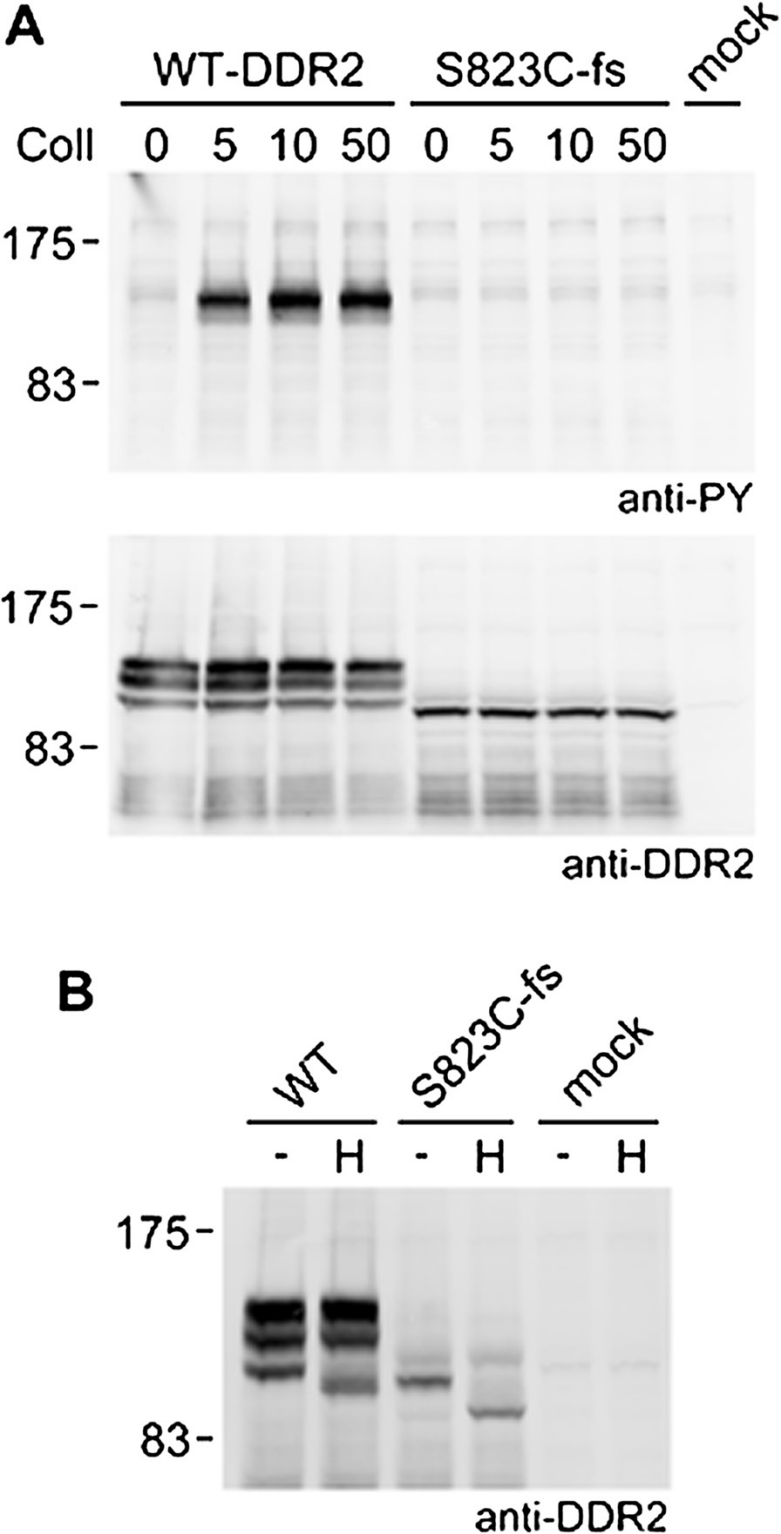
Defective cellular trafficking causes loss of collagen-induced signalling for the DDR2-S823Cfs* mutant. Full-length untagged wild-type DDR2 or S823Cfs* mutant were transiently expressed in HEK-293 cells. **(A)** Cells were stimulated for 90 min at 37°C with rat tail collagen I at the indicated concentrations (in μg/ml). Cell lysates were analyzed by SDS–PAGE and Western blotting. The blots were probed with anti-phosphotyrosine (anti-PY) monoclonal antibody 4G10 (upper blot) or polyclonal anti-DDR2 antibodies (lower blot). **(B)** Cell lysates were treated with Endoglycosidase H (H) or left untreated (-) for 3 h at 37°C and analyzed by SDS–PAGE and Western blotting. The blot was probed with polyclonal anti-DDR2 antibodies. The positions of molecular markers (in kDa) are indicated. The experiments were carried out twice with very similar results.

Due to glycosylation, DDR2 is often detected as a mixture of 3–4 bands between 125 kDa and 130 kDa [22,39,40], with the higher molecular weight forms representing heavily glycosylated mature protein and the smaller molecular weight bands corresponding to immature, partially glycosylated forms. In Western blot of lysates from HEK-293 cells overexpressing the S823Cfs* mutant, only the lowest molecular weight form was detected which was also found to migrate faster than the corresponding band in the wild type (Figure 4A, lower panel). The highest and intermediate molecular weight forms were not observed for the mutant while the wild type DDR2 was detected as a mixture of three bands including the highest and intermediate molecular weight forms. Based on previous reports, the lower molecular weight form of the mutant is presumably the ER retained partially glycosylated folding intermediate. This was further supported by deglycosylation of the mutant protein with Endoglycosidase H (Endo H), which specifically removes oligosaccharides of the high mannose and hybrid (pre-Golgi) forms, but not complex carbohydrate structures attained in the Golgi. Figure 4B shows the Endo H treated protein extracts from wild type and mutant over-expressing cells. The mutant protein was found to be sensitive to Endo H as was the lowest molecular weight wild type form. Deglycosylation caused both the forms to resolve as lower molecular weight bands. The higher molecular weight forms of the wild type DDR2 were found resistant to Endo H treatment. This confirms the ER localization of the mutant which was also evident from the confocal images (Figure 3).

Only the completely glycosylated and mature forms of DDR2 of the highest molecular weight (130kDa) is reported to reach the cell surface and get activated by collagen [22,39,40], which is a prerequisite for DDR2 receptor-mediated signaling. Upon collagen binding, DDR2 undergoes autophosphorylation at tyrosine residues in the cytoplasmic domain. HEK-293 cells expressing wild type or mutant DDR2 were treated with collagen as described in the Methods section, and tyrosine phosphorylation was analyzed by Western blotting using anti-phosphotyrosine (PY) antibodies. In contrast to wild type DDR2, which, as expected, showed a strong collagen-induced phosphorylation signal, no phosphorylation signal was detected for the S823Cfs* mutant (Figure 4A). These data strongly suggest that the mutation has led to loss of the DDR2 protein *in vivo* function.

## Discussion

We report here the clinical findings and radiological features of two siblings with SMED-SL and a novel DDR2 homozygous mutation. In addition to exhibiting the typical clinical and radiological features consistent with SMED-SL, our patients had some phenotypic variations between them. Progressive visual impairment and bilateral optic atrophy were observed in one of the patients. Abnormal eye movements and bilateral optic atrophy had been described before in SMED-SL [4]. The eye phenotype could be attributed to the fact that DDR2 is a plasma membrane RTK that functions as a collagen receptor to collagen II, a fibrillar collagen found in cartilage and the vitreous humor of the eye. Importantly the eye is made up of 80% collagen. Although the involvement of DDR2 in pathophysiologies related to the eye is not characterized, there is molecular evidence that DDR1 and DDR2 mRNAs and proteins are expressed in corneal epithelial cells, keratocytes and endothelial cells [41].

The disease-causing frame-shift mutation reported here is located on exon 18 of the *DDR2* gene, which is the last coding exon. The frame-shift causes an amino acid change of S823C and premature termination of translation, two amino acid residues downstream of the site of mutation. Premature termination codons appearing at the last coding exons of genes are found to be clinically relevant in several monogenic diseases. Transcripts of genes carrying premature termination codons located at least 50 nucleotides upstream of the last intron, are targets of an mRNA surveillance mechanism known as nonsense mediated decay (NMD). But mRNAs carrying the mutation in the last exon escape this mechanism which results in translation of truncated proteins which may exert a dominant negative effect even in the heterozygous carriers of the mutant allele [38]. Recently it was shown that Hajdu-Cheney syndrome, a rare skeletal disorder, is caused by heterozygous mutations clustering to the last exon of the *NOTCH* 2 gene, which code for a truncated protein that acts in a gain-of-function manner [42]. In a frame-shift mutation reported in *DDR1* gene, DDR1d, which is predicted to result in a membrane anchored, kinase deficient receptor, the mutant did not exert a dominant negative inhibition on the normal allele [43]. To establish a dominant negative effect for the novel DDR2 mutant, more detailed clinical, molecular and cellular analysis of the affected family is required.

The disease-causing mutation affects amino acid residues in the tyrosine kinase domain of DDR2. All kinases adopt a very similar structure [44] and hence loss of 32 amino acids from the DDR2 C-terminus is likely to affect protein stability, folding and plasma membrane targeting. Most kinases have a C-terminal tail, but in the case of DDR1 and DDR2, the catalytic kinase domains are followed by very short C-terminal tails. A crystal structure of the kinase domain of DDR1 shows that the C-terminal amino acids that are commonly assigned to a C-terminal ‘tail’ are actually part of an α helix in DDR1, which tightly interacts with the rest of the domain [45]. Therefore in the highly similar DDR2, loss of the 32 amino acids from the tightly folded C-terminus is likely to cause misfolding of the kinase domain and subsequent retention in the ER. Moreover, the high molecular weight form of DDR2 has been reported to be inherently unstable and dependent largely on glycosylation for efficient trafficking to the cell membrane [40]. We show here that the S823Cfs* mutant is retained in the ER and fails to reach the plasma membrane. As expected, the wild type protein localized predominantly to the plasma membrane, and a fraction of the wild type protein was observed also in the ER. Biochemical analysis of the mutant and wild type protein confirmed their intracellular distribution. The predominant form of mutant protein expressed in cells is found to be the immature lower molecular weight forms characteristic of ER retained folding intermediates. The wild type DDR2 exists as a mixture of mature high molecular weight and lower molecular weight intermediate forms. Further, the low molecular weight Endo H sensitive S823Cfs* mutant was not phosphorylated in response to collagen activation. Only the cell surface expressed DDR receptors bind to collagen, which induces phosphorylation of tyrosine residues in the cytoplasmic domain and downstream signalling [11]. Our results indicate that the S823Cfs* mutation results in an ER retained protein which is inactive *in vivo* upon adding collagen to the culture media. This is in agreement with our previous findings that mutations affecting the kinase domain of DDR2 result in misfolding and defective trafficking of the protein [7]. Our results also indicate that as predicted from the crystal structure of DDR1, the C-terminal amino acids are crucial for the stability of DDR2.

We have elucidated previously that missense mutations occurring in *DDR2* cause loss of function of the protein through two mechanisms, namely retention of the mutant proteins in the ER and loss of ligand binding activity [7]. The ER has a sophisticated quality control system that ensures trafficking of only mature proteins in their native conformation. Misfolded proteins that fail to conform to the ER quality control are retained in the ER and subsequently targeted for degradation by the ubiquitin/proteasomal machinery and have been implicated in the pathogenicity of many congenital disorders [31]. Recently it has been shown in a transgenic mouse model of growth plate dysplasia, that chronic ER stress is sufficient to cause decreased chondrocyte proliferation and reduction in bone growth, without inducing any alterations to the architecture and organization of cartilage extracellular matrix [30]. Chronic ER stress due to persistent accumulation of misfolded proteins has also been proposed to be involved in the development of other conditions including diabetes in some cystic fibrosis patients [46]. Concurrent to this hypothesis, in SMED-SL patients carrying *DDR2* mutations encoding plasma membrane expressed variants, the clinical symptoms were reported to be less severe than in patients carrying *DDR2* mutations encoding ER retained variants [7].

Spondylo-meta-epiphyseal dysplasia is a severe form of dwarfism with serious complications and for which no effective treatment is available. A previous study [5] suggested that SMED-SL should be included in the list of genetic disorders causing death. In genetic diseases caused by the impaired function of a single gene, various interconnected pathways are affected, making the identification of relevant pharmaceutical targets difficult. Accurately defining the consequences of underlying mutations is a challenge and prerequisite to envision targeted therapies to congenital diseases. We have described here a novel disease-causing mutation in SMED-SL, which results in defective intracellular targeting and therefore loss of in vivo ligand induced phophorylation of the DDR2 protein.

## Conclusions

This study documents the clinical findings and radiological features of two siblings with SMED-SL and a novel homozygous truncating mutation in the *DDR2* gene. The novel mutation affects the intracellular trafficking of DDR2 and consequently collagen induced activation of the receptor. In addition, our findings illustrates that the C-terminal amino acids are crucial for the stability of the DDR2 protein, since the novel mutation leads to loss of the C-terminal 32 amino acids of the protein.

## Competing interests

The authors declare no competing interests in the preparation or publication of the data in this manuscript.

## Authors’ contributions

BRA, LA and BL were responsible for the project conception and design of experiments. AJ performed genetic analysis, PK and HX performed confocal microscopy, biochemical assays and generated the figures. AAK, AAS, AG, MAA and LAA conducted clinical evaluations and compiled data. PK, LA, BRA, BL and HX wrote and edited the manuscript. All authors read and approved the final manuscript.

## Pre-publication history

The pre-publication history for this paper can be accessed here:

http://www.biomedcentral.com/1471-2350/15/42/prepub
